# DNA barcoding of Austrian terrestrial isopods (Crustacea: Isopoda: Oniscidea) reveals potential cryptic diversity

**DOI:** 10.1371/journal.pone.0352446

**Published:** 2026-07-06

**Authors:** Anna-Chiara Barta, Luise Kruckenhauser, Hedda Büttner, Matthäus Greilhuber, Klaus Hasenhütl, Stephan Koblmüller, Lukas Kremp, Oliver Macek, Hanna Sommer, Nikolaus U. Szucsich, Martin Schwentner

**Affiliations:** 1 Natural History Museum Vienna, 3rd Zoological Department, Vienna, Austria; 2 Department of Evolutionary Biology, University of Vienna, Vienna, Austria; 3 Natural History Museum Vienna, Central Research Laboratories, Vienna, Austria; 4 Department of Functional and Evolutionary Ecology, University of Vienna, Vienna, Austria; 5 Hasenhütl Consulting, Graz, Austria; 6 Institute of Biology, University of Graz, Graz, Austria; 7 Department of Medicine III, Medical University of Vienna, Vienna, Austria; University of Florida Tropical Research and Education Center, UNITED STATES OF AMERICA

## Abstract

Terrestrial isopods or Oniscidea are a diverse and ecologically important taxon. Austria has a relatively high species diversity with over 60 known species. In this study, we aim to provide DNA barcode reference data for the Austrian Oniscidea and to update the species inventory, which was last reviewed nearly 50 years ago. Although the focus was on freshly collected specimens, we also successfully sequenced historic museum specimens, thereby testing different museomic techniques including Sanger-based and Illumina-based methods. In total, 533 COI barcodes were generated, representing 49 of the 64 species and subspecies reported from Austria (= 76% of the species diversity), including two species not previously reported: *Philoscia muscorum muscorum* and *P. affinis affinis*. Several species exhibited large intraspecific genetic distances and the best-scoring ASAP partition (with a threshold of 6.2%) split the 49 studied species into 59 mOTUs. Even the more conservative partition with a threshold of 9.8% suggested the presence of further hitherto unrecognized species. One example is *Ligidium germanicum*, where we were able to demonstrate minor but consistent morphological differences in the shape of the endopod of pleopod II. COI data of historic specimens was successfully obtained. Sanger-based approaches using mini-barcodes in combination with a prior DNA repair step were more cost and labor effective than Illumina-based genome skimming approaches. This COI barcoding data plays an important role in bridging gaps in the representation of rare soil species, thus supporting the Austrian Barcode of Life Initiative (ABOL) and providing important reference data for future biodiversity and monitoring studies.

## Introduction

In the face of climate change and the biodiversity crisis, the comprehensive documentation of species diversity and distribution has become increasingly urgent [1]. This need is particularly pressing for soil communities, for which baseline data remain scarce [2]. DNA metabarcoding offers the possibility to quickly assess the diversity of soil communities [3–5]. However, the successful application of DNA barcoding, whether for individual specimen identification or for community-level surveys, requires well curated reference sequences (for Metazoa usually a fragment of the DNA barcoding gene COI) in the respective databases (i.e., GenBank or BOLD). For many key taxa, the reference coverage is incomplete and often restricted to only a subset of known species [5–7]. Terrestrial isopods (Oniscidea) represent a prime example. In Austria, the Austrian Barcode of Life (ABOL) initiative ([href:http://www.abol.ac.at]www.abol.ac.at) aims to record Austrian animal, plant and fungal biodiversity using an integrative approach that incorporates DNA barcoding as a standardised method [8].

**Fig 1:**
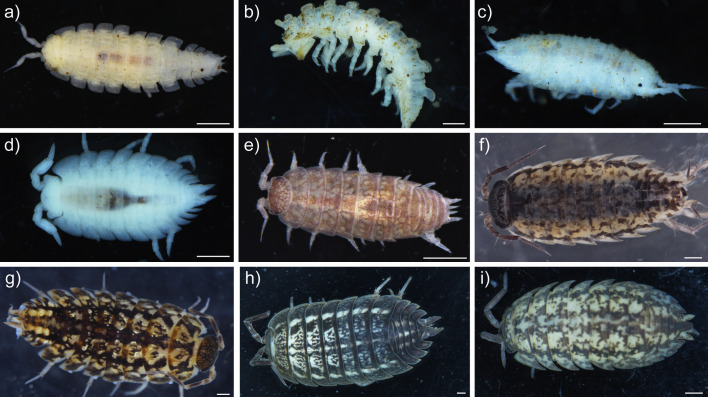
Examples of Austrian Oniscidea. **a)** *Haplophthalmus austriacus* (NHMW-ZOO-CR-29993), **b)** *Calconiscellus karawankianus* (NHMW-ZOO-CR-28416), **c)** *Androniscus roseus* (NHMW-ZOO-CR-29089), **d)** *Platyarthrus hoffmannseggi* (NHMW-ZOO-CR-28044), **e)** *Trichoniscus steinboecki* (NHMW-ZOO-CR-30047), **f)** *Ligidium hypnorum* (NHMW-ZOO-CR-27973), **g)** *Lepidoniscus minutus* (NHMW-ZOO-CR-26602), **h)** *Trachelipus rathkii* (NHMW-ZOO-CR-28274), **i)** *Porcellium conspersum* (NHMW-ZOO-CR-27979). Scale bars: 500 µm. Photos NHMW.

Oniscidea, commonly known as woodlice, is a diverse and ubiquitous, and ecologically important component of terrestrial ecosystems (Fig 1). With >3,600 species globally [9], oniscidean isopods occur in virtually all terrestrial ecosystems and they play a key role as decomposers in soil communities. The monophyly of Oniscidea is debated (e.g., [10], however, the most extensive molecular phylogenomic study of Isopoda strongly supported oniscid monophyly [11]. Austria hosts a relatively species-rich fauna of terrestrial isopods including many species widespread in central and northern Europe as well as southern alpine species probably stemming from south-eastern Europe [12] and several endemic species [13]. The austrian Oniscidea fauna has been studied in great detail in the early and mid 20th century (reviewed in [12,14–16], but has since then received very little attention; noteworthy exceptions being a recent list on Austrian endemic species [13] and local faunal inventories [17–21]. In 1951, Strouhal listed 58 species and subspecies for Austria in his very comprehensive overview [12]. He updated this count in 1968 [14], when he counted 64 species and subspecies. According to Strouhal, between 1951 and 1968 three (sub)species had to be disregarded as they had become junior synonyms of other Austrian (sub)species (*Trichoniscus ostarrichius* Strouhal, 1947, *Haplophthalmus perezi* Legrand, 1942 and *Lepidoniscus minutus ericarum* Verhoeff, 1908), four had been newly reported for Austria (*Trichoniscus pusillus provisorius* Racovitza, 1908 [now *T. provisorius* Racovitza, 1908], *Trichoniscus carniolicus* Strouhal, 1939, *Trichoniscus pygmaeus* Sars, 1899 and *Porcellio dilatatus dilatatus* Brandt, 1833) and five had been newly described. The latter were not specifically listed but probably referred to *Haplophthalmus mariae* Strouhal, 1953, *Oroniscus mandli* Strouhal, 1958, *Trichoniscus illyricus carinthiacus* Strouhal, 1958, *Trichoniscus scheerpetzi* Strouhal, 1958, *Trichoniscus styricus* Strouhal, 1958. It appears that Strouhal [14] overlooked *Haplophthalmus montivagus* Verhoeff, 1941, which he had reported from Austria [22], which would have raised the number of Austrian Oniscidea species to 65. Schmölzer [16] followed up on this list and added a few more subspecies, which either had been abandoned previously or which had not been reported by other authors. As none of these are currently accepted (e.g., [9]), we do not include them here either. Since then, one putatively invasive species has been newly reported from Austria, namely *Armadillidium arcangelii* Strouhal, 1929 [23] (see Table 1). According to Schmölzer [24] and Schmalfuss [9], the Austrian and other European records of *Protracheoniscus asiaticus* are in fact misidentified records of *P. major*; we follow this suggestion and use *P. major* in the following.

**Table 1:** Updated overview of the Oniscidea species occurring in Austria. The list is largely based on Strouhal [14] and Schmölzer [16] and includes the species first reported herein. Species names were updated based on the currently accepted taxonomy. If DNA barcodes were generated, the respective BOLD BIN number(s) and the number of mOTUs for each of the three main ASAP partitions are provided per species (numbers set in bold indicate new BIN numbers). *three *Trachelipus* sequences were short and non-overlapping, therefore ASAP and the phylogenetic analyses could not properly analyze these. A comparison to the full-length data from Raupach et al. [25] (S2 Fig) shows that our *L. nodulosus* sequence was clearly assigned to *T. nodulosus*, whereas the two *T. arcuatus* sequences clearly differed from these and from each other.

Species	Number mOTUs ASAP4.8	Number mOTUs ASAP6.2	Number mOTUs ASAP9.8	BOLD BIN	Comment
*Androniscus roseus* (C. Koch, 1838)	1	1	1	BOLD:ADE9981	
*Androniscus stygius* (Némec, 1897)					[12] with two subspecies: *A. s. tschameri* and *A. s. kesselyáki*
*Androniscus subterraneus* (Carl, 1906)					
*Armadillidium arcangelii* Strouhal, 1929	1	1	1	BOLD:AGO4774; BOLD:AGO4776	first reported for Austria by Noël et al. [23]
*Armadillidium carniolense* Verhoeff, 1901	1	1	1 (with *A. opacum*)	**BOLD:AGY9977; BOLD:AGY9980**	
*Armadillidium carynthiacum* Verhoeff, 1939					
*Armadillidium nasatum* Budde-Lund, 1885					
*Armadillidium opacum* (C. Koch, 1841)	1	1	1 (with *A. carniolense*)	BOLD:AAV4460	
*Armadillidium pictum* Brandt, 1833	1	1	1	BOLD:AAV9852	
*Armadillidium versicolor* Stein, 1859	1	1	1	**BOLD:AGY9981; BOLD:AGY9982**; BOLD:AAV9853	
*Armadillidium vulgare* (Latreille, 1804)	1	1	1	**BOLD:AGY9979; BOLD:AGZ1239;** BOLD:AAE6611; BOLD:AAH4111; BOLD:AAU1529; BOLD:AEK7048; BOLD:AFF2512	
*Armadillidium zenckeri* Brandt, 1833	1	1	1	**BOLD:AGY9978**	
*Calconiscellus karawankianus* (Verhoeff, 1908)	1	1	1	**BOLD:AGZ1919; BOLD:AGZ1920**	
*Cylisticus convexus* De Geer, 1778	2	1	1	**BOLD:AGY7769**; BOLD:AAU8054	
*Haplophthalmus austriacus* Verhoeff, 1941	1	1	1	**BOLD:AGY5228**	in [12] erroneously as *H. legrandi*
*Haplophthalmus danicus* Budde-Lund, 1880	1	1	1	BOLD:AAU3256	
*Haplophthalmus mariae* Strouhal, 1953					
*Haplophthalmus mengii* (Zaddach, 1844)	1	1	1	BOLD:ADM7489	
*Haplophthalmus montivagus* Verhoeff, 1941					
*Hyloniscus adonis* Verhoeff, 1927	1	1	1	**BOLD:AGZ2108; BOLD:AGZ2109**	one additional mOTU recovered, which is from Slovenia
*Hyloniscus riparius* (C. Koch, 1838)	1	1	1	BOLD:AAV6495	
*Lepidoniscus minutus minutu*s (C. Koch, 1838)	1	1	1	BOLD:AAV7783	usually reported as var. *pannonicus*
*Lepidoniscus pruinosus pruinosus* Carl, 1908					
*Ligidium germanicum* Verhoeff, 1901	2	2	2	**BOLD:AGY2821**; BOLD:AAE0735; BOLD:AED6140	
*Ligidium hypnorum* (Cuvier, 1792)	1	1	1	BOLD:AAF5619	
*Mesoniscus alpicola* (Heller, 1858)	2	2	2	**BOLD:AGY2729; BOLD:AGY2730**	
*Oniscus asellus* Linnaeus, 1758	1	1	1	BOLD:ADK9123; BOLD:ADM8116	
*Oroniscus mandli* Strouhal, 1958	1	1	1	**BOLD:AGY2317**	
*Philoscia affinis affinis* Verhoeff, 1908	1	1	1	**BOLD:AGZ1797**; BOLD:AAY1058	not previously reported for Austria
*Philoscia muscorum muscorum* (Scopoli, 1763)	1	1	1	BOLD:AAH4103	not previously reported for Austria
*Platyarthrus hoffmannseggii* Brandt, 1833	3	2	2	**BOLD:AGY1979**; BOLD:AAV8050	
*Porcellio laevis* Latreille, 1804	1	1	1		
*Porcellio scaber* Latreille, 1804	2	2	1	BOLD:AAC3755; BOLD:ADM8147; BOLD:ADV3050	
*Porcellio spinicornis* Say, 1818	1	1	1	BOLD:ADI3596; BOLD:AGA9677	
*Porcellio dilatatus* Brandt in Brandt & Ratzeburg, 1831					
*Porcellionides pruinosus* (Brandt, 1833)	1	1	1	BOLD:AAH4110	
*Porcellium collicola* (Verheoff, 1907)	1	1	1	BOLD:AEO7189	
*Porcellium conspersum* (C. Koch, 1841)	1	1	1	BOLD:AAV7038	
*Porcellium fiumanum fiumanum* (Verhoeff, 1901)	1	1	1 (with *P. f. salisburgense*)	BOLD:AGY1068	
*Porcellium fiumanum salisburgense* Verhoeff, 1928	1	1	1 (with *P. f. fiumanum*)	BOLD:AAV7039	
*Porcellium recurvatum* (Verhoeff, 1901)	1	1	1	**BOLD:AGY1070**	in [12] as *P. graevei*
*Protracheoniscus franzi* Strouhal, 1948	1	1	1	no BIN assigned (too short)	
*Protracheoniscus hermagorensis* Verhoeff, 1927	1	1	1	**BOLD:AGY1069**	
*Protracheoniscus major* (Dollfus, 1903)					previously reported as *P. asiaticus*, following Schmölzer [24] the correct identification is *P. major*
*Protracheoniscus politus* (C. Koch, 1840)	1	1	1	BOLD:ADK9732	previously listed as *P. amoenus* (now synonymized)
*Tachysoniscus austriacus* (Verhoeff, 1908)	1	1	1	**BOLD:AGY1262**	
*Trachelipus arcuatus* (Budde-Lund, 1885)	2 *	2 *	2 *	no BIN assigned (too short)	previously also the now synonymized *T. pseudoratzeburgi apenninorum* was listed
*Trachelipus nodulosus* (C. Koch, 1838)	*	*	*	**BOLD:AGY1067**	
*Trachelipus rathkii* (Brandt, 1833)	1	1	1	**BOLD:AGY1066; BOLD:AGZ1571; BOLD:AGZ1572; BOLD:AGZ1573; BOLD:AGZ1574**; BOLD:AAH4100; BOLD:AAH4102; BOLD:AAH4109; BOLD:ADM8088	
*Trachelipus ratzeburgii* (Brandt, 1833)	3	3	1	**BOLD:AGY1063; BOLD:AGY1064; BOLD:AGY1065**; BOLD:AAV6691	
*Trichoniscus alemannicus* (Verhoeff, 1917)					
*Trichoniscus carniolicus* Strouhal, 1939	1	1	1	**BOLD:AGY1018**	
*Trichoniscus crassipes* Verhoeff, 1939					
*Trichoniscus illyricus carinthiacus* Strouhal, 1958					
*Trichoniscus karawankianus* Verhoeff, 1939					
*Trichoniscus muscivagus* Verhoeff, 1917	1	1	1	**BOLD:AGY1019**	
*Trichoniscus nivatus* Verhoeff, 1917					
*Trichoniscus noricus* Verhoeff, 1917	2	2	2	**BOLD:AGY1015**; BOLD:ADL4642	
*Trichoniscus provisorius* Racovitza, 1908	1	1	1	**BOLD:AGY1020**	
*Trichoniscus pusillus* Brandt, 1833	2	2	2	BOLD:AAN7523; BOLD:AAZ1993	
*Trichoniscus pygmaeus* G.O. Sars, 1898	1	1	1		
*Trichoniscus scheerpeltzi* Strouhal, 1958	2	1	1	**BOLD:AGY1011; BOLD:AGY1013**	
*Trichoniscus steinboecki* Verhoeff, 1931	3	3	2	B**OLD:AGY1010; BOLD:AGY1012; BOLD:AGY1014; BOLD:AGY1016**	
*Trichoniscus styricus* Strouhal, 1958					

Despite their ecological importance and high diversity, many species have not been studied genetically yet, and thus no reference DNA barcodes are available. The most comprehensive European DNA barcoding effort on terrestrial isopods to date targeted the German fauna [25], generating COI barcode sequences for 38 of the 49 species known from Germany. Apart from providing DNA barcodes, the study indicated the presence of highly divergent genetic lineages within several of the studied species. This may indicate the presence of additional species. Alternatively, also other factors like NUMTS (nuclear mitochondrial DNA segments), infections with the endosymbiont *Wolbachia* or historic subdivision may have caused high intraspecific genetic diversities [25].

Producing a reliable DNA barcode reference library depends on the accurate morphological identification of the sequenced specimens. However, species identification in terrestrial isopods is often challenging. In many cases, the main characteristics used to define and identify species are based on males (i.e., shape of 7th pereiopod or the shape of pleopods I and II), rendering the identification of females or juveniles difficult or impossible. For many of the small-bodied Trichoniscidae, the preparation of the pleopods can be challenging and is at least partially destructive. The lack of good diagnostic characters for females is particularly problematic as in many species populations are often highly female-biased (e.g., [26,27]). The underlying causes have not always been studied, but infections with the endosymbiotic bacterium *Wolbachia* are widespread in terrestrial isopods and *Wolbachia* infections can cause feminization [28–31].

DNA barcoding studies usually focus on freshly collected specimens, mainly because their DNA is of higher quality, making the generation of DNA barcodes easier. However, museum collections can also play an important role in the establishment of DNA barcode reference databases [32]. On the one hand, they hold vast collections, including many rare and difficult to access species, which otherwise could hardly be included in any study. On the other hand, they also hold historic taxonomic knowledge as many of the historic specimens have been determined by various taxonomic experts, many of which are now retired or deceased. Utilizing this knowledge is crucial, especially in light of the increasing taxonomic impediment [33]. But historic DNA (hDNA) from museum collections is often of low quality: low DNA amounts, highly fragmented and much of it single-stranded. Prior fixation with formalin can further enhance these problems by also adding protein-DNA cross-links, deamination, etc. [34]. To overcome these limitations, a range of methods have been developed. For example, by trying to repair some of the common DNA damages (like deamination, single strand nicks etc), by utilizing primers that amplify shorter DNA barcode regions (so called mini-barcodes; e.g., [35,36], amplifying and sequencing the barcoding region in several short overlapping fragments (e.g., [37,38]), or by using genome skimming [39,40]. For the latter, additional protocols have been developed to transform single-stranded DNA (ssDNA) into double-stranded (dsDNA) to also utilize the more abundant ssDNA in the genomic library preparation (e.g., [41]). Collectively, these approaches form the emerging field of museomics – genomic research using museum material (reviewed in [42–45].

The goals of the present study were fourfold: i) to generate DNA reference barcodes of Austrian Oniscidea as part of the Austrian Barcode of Life Initiative, ii) to update the checklist of Austrian terrestrial isopods, iii) to assess the degree of intra- and interspecific genetic divergence, and iv) to test museomic techniques to facilitate the integration of historic museum samples.

## Materials and methods

### Species list

To obtain an overview of the Austrian terrestrial isopod fauna, we updated the lists provided by Strouhal [12,14] and Schmölzer [16], which are the most detailed lists for Austria. As these include species that have since been synonymized or subspecies that have been raised to species, the validity of all species was checked against the list by Schmalfuss [9] and WoRMS (accessed 06/2025). The list was further complemented with other reports (e.g., [23]) and our own data.

### Sample preparation of fresh material

**Fig 2:**
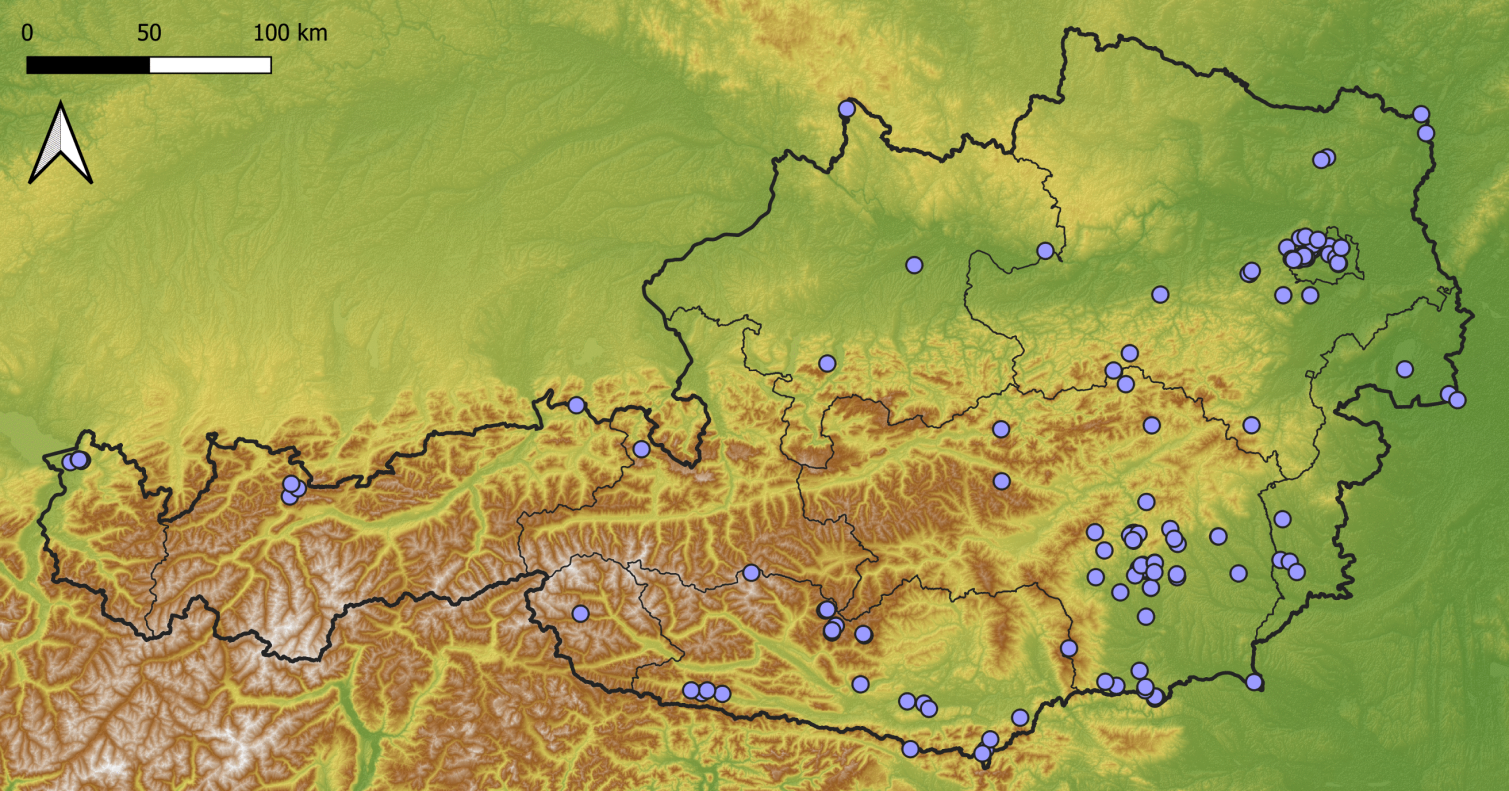
Map. Sampling localities of all recently collected and successfully DNA barcoded Austrian specimens are shown. Blue = sampling points. Thick black line = country border of Austria. Narrow black lines = federal state borders. Historical localities without available coordinates are not depicted on the map. Map based on Copernicus GLO-30 Digital Elevation Model downloaded from OpenTopography (https://portal.opentopography.org/raster?opentopoID=OTSDEM.032021.4326.3), boundary source: geoBoundaries, Federal Office for Metrology and Survey, Austria [47].

Terrestrial isopods were collected between 2021 and 2025 from various habitats across Austria and preserved in 80% methanol-denatured ethanol (Fig 2; S1 Table). In addition, five individuals collected in neighboring Slovenia were included as well. Species identification was based mainly on Schmölzer [24] and the interactive key in https://bodentierhochvier.de as well as various original descriptions of species. As in several genera only males can be unambiguously identified to species, we largely employed a reverse taxonomic approach (following [46]), in which specimens are first clustered based on their DNA sequence data into putative species (molecular Operational Taxonomic Units = mOTUs) and then these clusters are assigned a species name based on the identification of the associated individuals. Specimens that themselves cannot be identified to species level (i.e., females, juveniles, damaged) are thus assigned the species name by genetic similarity. A map of all recent sampling localities (historic localities are not shown as their exact position are often vague) was made using QGIS version 3.28.14. The Copernicus GLO-30 Digital Elevation Model was downloaded from OpenTopography (https://portal.opentopography.org/raster?opentopoID=OTSDEM.032021.4326.3), boundary source(s): geoBoundaries, Federal Office for Metrology and Survey, Austria (https://www.geoboundaries.org/index.html) [47].

DNA was extracted from a single leg each. For very small-bodied individuals (mainly Trichoniscidae) a section of the mid-body was removed with sterile needles. The tissues were incubated in 10 μl Proteinase K (GeneOn) and 90 μl ATL lysis buffer (DNeasy Blood and Tissue kit, Qiagen) at 56°C for 2–4 hours. DNA was purified using 100 μl Ampliclean (Nimagen) magnetic beads, following the manufacturer’s recommendations. DNA was eluted in 40 μl nuclease-free H_2_O. The extracted DNA samples were stored at −20°C.

A fragment of the cytochrome c oxidase subunit I (COI) gene, commonly used as a DNA barcode region for animal species, was PCR amplified using the LCO1490/HCO2198 primer pair (5’-GGTCAACAAATCATAAAGATATTG-3’/5’-TAAACTTCAGGGTGACCAAAAAATCA-3’; [48]). If these did not yield a PCR product, a shorter 316 bp fragment (so called mini-barcode) was amplified using the primer pairs BF1 (5’-ACWGGWTGRACWGTNTAYCC-3’) and BR2 (5’-TCDGGRTGNCCRAARAAYCA-3’; [49]) or BF1 and HCO2198. 15µl PCR reactions included 0.05µl DreamTaq DNA polymerase (Thermo Scientific), 1.5µl 10x DreamTaq Green Buffer, 0.3µl dNTPs (10µM each), 1.5µl each primer (10mM), 8.65µl H_2_O and 1.5µl DNA extract. If amplification failed repeatedly, another taq-polymerase was employed: 7.5µl 2x QIAGEN Multiplex PCR Master Mix (Qiagen), 1.5µl of each primer (10mM each), 2 µl DNA extract and 2.5µl H_2_O were combined and run with following PCR cycling program: 95°C for 15 min, 35 cycles of 30 s at 95°C, 45 s at 46°C and 1 min at 72°C and a final elongation of 5 min at 72°C. PCR products were visualized on 1.5% agarose-TBE gels. Prior to sequencing, primers and dNTPs were inactivated with 0.6 µl shrimp alkaline phosphatase (rSAP, NEB) and 0.3 µl exonuclease I (NEB) (incubated for 15 min at 22°C and 20 min at 85°C). Cleaned PCR products were bidirectionally sequenced using the respective PCR primers with the Sanger sequencing service of Macrogen. The resulting electropherograms were assembled, manually curated and aligned with Geneious PrimeⓇ 2025.0.3.

### Ethics statement

Specimens were collected under the following collection permits: RU5-BE-64/023–2022 (Lower Austria), 2024-009.459-1/6 (Burgenland), A4/NR.AB-10007-12-2022 (Burgenland), MA22–302672/2024 (Vienna), ABT13–523696/2023–9 (Styria), VK3-NS −2790/2024 (005/2024) (Carinthia), FE3-NS-3710/2024 (005/2024) (Carinthia). This work did not require ethical approval from a human subject or animal welfare committee.

### Sample preparation of historic material

From the historic collection of the Natural History Museum of Vienna, Austrian specimens identified by former Oniscidea expert Hans Strouhal were selected. Strouhal was the curator of the crustacean collection at the Natural History Museum Vienna from 1946 to 1962 with a strong focus on terrestrial isopods. Animals collected before 1900 were prioritized to avoid fixation in formaldehyde (the original fixative is rarely documented for the historic material), which further reduces the quality of historic samples due to issues like deamination of cytosine to uracil, crosslinks or single strand breaks [34]. 80 historic individuals were selected for DNA extraction.

DNA extraction of all historical samples was performed in the clean room of the Natural History Museum Vienna. Through UV radiation and filter systems, this facility enables contamination-free DNA extraction, which is particularly important for museum material. The DNA extraction protocol for historical samples was similar to that used for fresh samples (see above). To account for the low DNA concentration in museum samples, a larger amount of tissue was utilized compared to the fresh material. Small specimens were placed whole in the extraction buffer and the remaining exoskeleton preserved after digestion. To compensate for the increased proportion of tissue, the amount of proteinase K and ATL lysis buffer was doubled and also the relative amount of magnetic beads increased (up to 2x) to 300μl to retain small DNA fragments as well. DNA was eluted in 20–30 µl H_2_O.

At first, PCR amplification via the standard primers for full length COI as well as mini-barcode primers BF1 and BR2 was tested as described above. As this failed on most instances, a DNA repair step with the NEBNext® FFPE DNA Repair v2 (E7360) module following the manufacturer’s instruction (except that all volumes were reduced by 50% to reduce costs) was tested for 21 DNA extracts. This kit is supposed to fix several typical problems of historic DNA (e.g., deamination, single-strand breaks, etc). Then the PCR with mini-barcodes was repeated and PCR products were Sanger sequenced as described above.

We further generated NGS Illumina libraries for genome skimming to obtain COI barcode sequences. Only dsDNA can be ligated to Illumina adaptors to construct genomic libraries. As a large fraction of hDNA is single-stranded, we tested a method that converts ssDNA to dsDNA prior to library construction for some samples. After DNA extraction, the dsDNA was quantified with the ThermoFischer Scientific® Qubit dsDNA HS Assay kit (Q32854) and ssDNA with the Qubit ssDNA Assay Kit (Q10212). Only for samples with sufficient DNA amounts library preparation commenced. First, potential DNA damages were repaired with the NEBNext® FFPE DNA Repair v2 module (see above) for each DNA extract. Library preparation was performed either directly using the repaired DNA or ssDNA was first converted into dsDNA using the ‘G-tailing’ protocol [41]. The G-tailing method adds a series of guanines to the ssDNA which then serves as a priming site for a C-primer to synthesize the second DNA strand [41]. 38.5 µl repaired DNA was denatured for 15 min at 96°C, then 5 µl 10x TdT buffer, 5 µl CoCl_2_ (2.5 nM), 0.5 µl Terminal Transferase (20 U/µl) and 1 µl dGTP (5mM) (all from New England Biolabs) were added and incubated for 30 min at 37°C and 10 min at 70°C to add approximately 6–8 guanine bases. After clean-up using Ampliclean magnetic beads, DNA was eluted in 18 µl H_2_O of which 16.5 µl were used in the following reaction. Second strand synthesis utilized 4.5 µl dNTPs (10mM each), 3 µl 10x NEBbuffer, 3 µl Klenow Fragment (5 U/µl), 3 µl primer (comprising 6 cytosine bases; 15 mM) and 16.5 µl of the G-tailed DNA and was incubated at 23 °C for 180 min and 75 °C for 20 min. The product was then cleaned-up with magnetic beads, with the elution volume being adapted to the respective library protocol that followed.

Library preparation was performed either with the NEBNext® Ultra™ II DNA Library Prep Kit for Illumina or the NEBNext® Ultra™ II FS DNA Library Prep Kit for Illumina (which has an additional fragmentation step at the beginning of the library preparation). We used half of the volumes of all reagents (including input DNA volumes) and otherwise followed the recommendation by the manufacturer. Adapters were stubby y-yoke adapters following Glenn et al. [50], to which a 5 bp barcode region had been added next to the ligation site of the adapter. The adapters gained full length during PCR amplification, which also added an index. This allowed a flexible indexing strategy, on the one hand by the barcodes (which always add the same unique barcode on both sides of the library and which represents the first 5 bp of each read) and on the other hand the two PCR primers, which adds the standard Illumina indices. This allowed multiplexing of several samples with a large number of unique barcode and index combinations. PCR amplification utilized the NEBNext Ultra II Q5 Master Mix included in the library preparation kits, setting up two PCR reactions of 10µl with 15 PCR cycles for each library to reduce amplification bias; these replicates were each pooled before beads clean-up and quantification. All clean-up steps were performed with Ampliclean magnetic beads. Prior to PCR amplification, 1.5-2x beads to library (or DNA) ratios were used to retain short DNA fragments, after PCR 1x beads to library ratios were used to remove primer dimers. Prior to sequencing and final pooling, all libraries were quantified with the Qubit 4 Fluorometer by Thermo Fisher Scientific and 4200 Tapestation D1000 tapes to assess fragment size distribution and to allow pooling at equal molarity. As some libraries had fragments >1000 bp, the final sequencing pool was run on Sage Science’s BluePippin DNA Size Selection System with a 1.5% agarose cassette retaining only DNA fragments between 250–600 bp prior to sequencing. Sequencing commenced with Genohub sequencing service with an Illumina HiSeq 4000. Illumina reads were trimmed with TrimGalore v. 0.6.2 (https://github.com/FelixKrueger/TrimGalore) with a minimum per base quality of 30. To retrieve COI sequences, reads were assembled with MitoFinder [51], providing all Isopoda whole mitochondrial genomes available on NCBI as references for gene identification.

### Species delimitation

All COI sequences were combined in a single alignment. The complete dataset has been submitted to BOLD (S1 Table; dx.doi.org/10.5883/DS-ISOBA). Austrian representatives of *Asellus aquaticus* (a non-oniscidean freshwater isopod) were included as an outgroup. To obtain estimates of putative species (=mOTUs), species delimitation was performed with ASAP (Assemble Species by Automatic partitioning; [52]) using the ASAP web service (https://bioinfo.mnhn.fr/abi/public/asap/asapold.html) employing *p*-distances and standard settings. To further test if the mOTUs or putative species delimited by ASAP are reciprocally monophyletic, a maximum likelihood tree was inferred using RAxML [53], employing the GTR + I + G substitution model and 1000 rapid bootstrap replicates. RAxML was run on http://usegalaxy.eu [54]. The tree was visualized with FigTree ver. 1.4.3 The goal of the phylogenetic analysis is to test the support of the mOTUS and species, not to assess phylogenetic relationships among taxa. With only a single and highly variable gene, the relationships of these evolutionary old taxa cannot be adequately reconstructed. Pairwise genetic distances were calculated in MEGA-X [55] using *p*-distances and pairwise deletion.

To compare our data to that of Raupach et al. [25], who provided the most comprehensive DNA barcode dataset for terrestrial Isopoda from central Europe, and to assess if the mOTUs recovered for the species were the same, we downloaded the corresponding COI dataset from NCBI. The data was aligned and a joint phylogenetic and molecular species delimitation analysis was performed with RAxML and ASAP as described above.

## Results

514 Sanger sequences were generated of freshly collected oniscids and five of the outgroup *A. aquaticus*. In the following species and mOTU counts *A. aquaticus* will not be included. In addition, 19 COI sequences were obtained from historic specimens (see below for details). The alignment did not include any indels or stop codons and was trimmed to a length of 552 bp.

**Fig 3:**
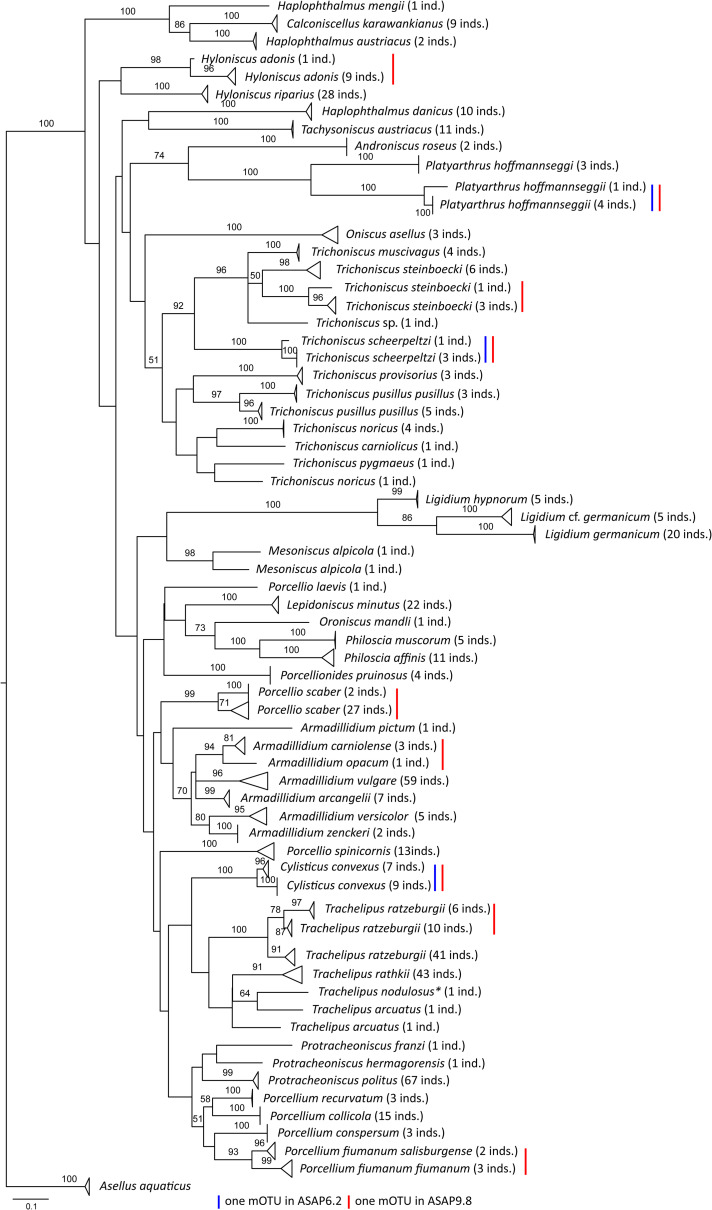
Maximum likelihood phylogenetic tree of Austrian Oniscidea based on COI. Nodes were collapsed to show putative species (mOTUs) suggested by ASAP with a threshold of 4.8% (ASAP:4.8). mOTUs collapsed with threshold values of 6.2% or 9.8% are indicated by blue and red bars, respectively. Only bootstrap support values >50 are shown. The uncollapsed tree with all branches can be found as S1 Fig.

ASAP provides several alternatives on how the COI data can be partitioned into mOTUs, depending on the employed threshold value. The best scoring ASAP partition (threshold 6.2%, ASAP score 7.0) delimited 59 mOTUs (Table 1; Fig 3). To contrast this, we also focus on two partitions with lower and higher threshold values, respectively. With a threshold of 9.8% (ASAP score 8.0) 51 mOTUs and with a threshold of 4.8% (ASAP score 20.5) 62 mOTUs are delimited; all mOTUs were monophyletic in the phylogenetic analysis (Table 1; Fig 3; S1 Fig). In the following, we will refer to these as ASAP4.8, ASAP6.2 and ASAP9.8 according to their threshold values. Notably, the automatically assigned Barcode Identification Number (BIN) by BOLD, which represents clusters of barcodes potentially representing species, resulted in >80 BINs (Table 1; S1 Fig). This number does not include another five species, whose barcode sequences were too short to be assigned a BIN. As this probably greatly overestimates actual species diversity, we will not discuss the BINs in greater detail.

These 51–62 delimited mOTUs correspond to 49 morphologically identified nominal (sub)species (Table 1). 36 of these nominal species corresponded to a single mOTU under all three ASAP thresholds. In some instances, a single nominal (sub)species included more than one mOTU, conversely, multiple nominal (sub)species were only rarely united into a single mOTU. One *Trichoniscus* mOTU could not unambiguously be assigned to a nominal species, because no males were available.

*Ligidium germanicum, Mesoniscus alpicola, Platyarthrus hoffmannseggii, Trichoniscus noricus, Trichoniscus pusillus* and *Trichoniscus steinboecki* were in all three ASAP partitions consistently delimited into two or three mOTUs each, whereas *Porcellio scaber, Hyloniscus adonis, Cylisticus convexus* and *Trachelipus ratzeburgii* were delimited into two or three mOTUs only in ASAP4.8 and partly ASAP6.2 (Fig 3; Table 1). Furthermore, *Porcellium fiumanum* was separated into two mOTUs, corresponding to *Porcellium fiumanum fiumanum* (Verhoeff, 1901) and *Porcellium fiumanum salisburgense* Verhoeff, 1928 in ASAP4.8 and ASAP6.2, but merged into a single mOTU in ASAP9.8. Similarly, *Armadillidium opacum* and *Armadillidium carniolense* were split into two mOTUs in ASAP4.8 and ASAP6.8, but assigned to a single mOTU in ASAP9.8.

The delimitation of *Trachelipus* species was complicated by the non-complete COI sequences of two historic specimens (Fig 3; S1 Fig). While one individual originally identified as *T. balthicus* (Verhoeff, 1907) (=*T. nodulosus*; specimen CR14264) had only the last ~200 bp of the COI sequence, the other specimen identified as *T. arcuatus* (Budde-Lundt, 1885) (specimen CR14367) has only the first ~350 bp. This resulted in an overlap of only ~20 bp, too little for the analyses to properly assess whether they belong to the same species or not. Both were clearly differentiated from another *T. arcuatus* specimen (originally identified as *T. pseudoratzeburgi apenninorum;* specimen CR14401), *T. rathkei* and *T. ratzeburgii.*

**Fig 4:**
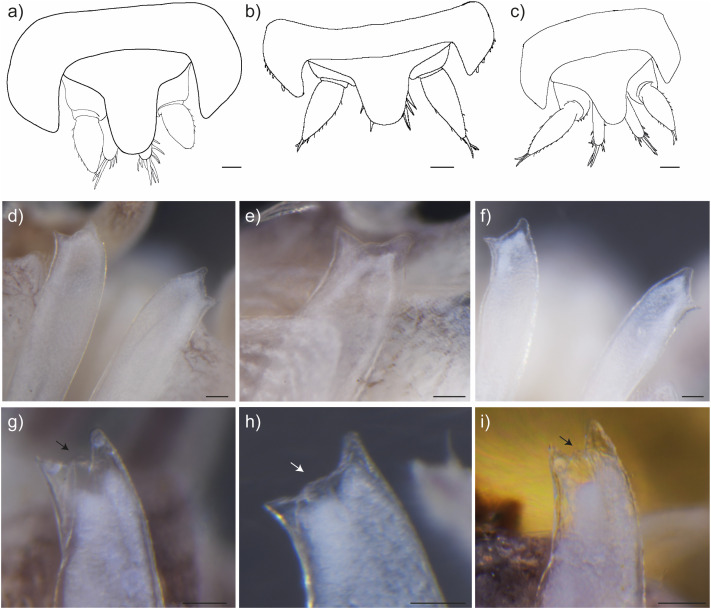
Morphological characters. **a)**-c) pleon and telson highlighting the relative length of the uropod endopod to the telson: **a)** *Porcellium recurvatum* (NHMW-ZOO-CR-28427); **b)** *Porcellium collicola* (NHMW-ZOO-CR-28318); **c)** *Porcellium collicola* (NHMW-ZOO-CR-28292); **d)**-f) tip of pleopod II endopod of *Ligidium germanicum*: **d)** NHMW-ZOO-CR-29992; **e)** NHMW-ZOO-CR-29883; **f)** NHMW-ZOO-CR-29920; **g)**-i) tip of pleopod II endopod of *Ligidium* cf. *germanicum*, arrows indicate the additional protrusion or hump in the center of the tip: **g)** NHMW-ZOO-CR-28396; **h)** NHMW-ZOO-CR-27943; **i)** NHMW-ZOO-CR-28395. Scale bars: **a)**-c): 100µm, **d)**-i) 50 µm. Photos NHMW.

In some instances, taxonomic identification was problematic following current keys, for example for *Porcellium collicola*. One important characteristic for the identification of *P. collicola* is the length of the uropod endopod, which is shorter than and covered by the telson. However, some individuals falling into the respective mOTU had distinctly longer uropod endopods (Fig 4). The species identity could be clearly settled by the shape of the male pleopod endopodite I, indicating that the length of the uropod endopod is not always a reliable character. The species identification of *P. recurvatum* was problematic as no males were available. Our species identification is based on the strong indentation of the posterior edge of the first pereion tergites in combination with the length of the uropod exopodites and the shape of the telson (Fig 4).

We recorded two species of *Philoscia*, *P. affinis affinis* Verhoeff, 1908 and *P. muscorum muscorum* (Scopoli, 1763), which had not previously been recorded from Austria. The introduced species *Armadillidium arcangeli* was found only in Vienna.

For most species with multiple intraspecific mOTUs, there was no or only limited correspondence between mOTUs and their geographic distribution. For example, while one mOTU (recovered in ASAP4.8 and ASAP6.2) of *T. ratzeburgii* was recorded only in Lower Austria, other specimens from Lower Austria belong to the respective other mOTU. Similarly, specimens from Vienna of *C. convexus* were present in both mOTUs of this species. A notable exception is *P. scaber*, where all specimens from far western Austria (Vorarlberg) constitute a separate mOTU.

The comparison to the dataset of Raupach et al. [25] showed that for most species, which were included in both datasets, the same mOTUs were recovered from Austria and Germany (S2 Fig). Noteworthy exceptions are *Mesoniscus alpicola* (here, the German sequence is clearly divergent form the two Austrian mOTUs), of *P. politus* and *T. rathkii* a second mOTU each was found only in Germany, two of the three *T. ratzeburgii* mOTUs were found only in Austria, and one of the *C. convexus* mOTUs was found only in Austria. Out of the three short and non-overlapping *Trachelipus* sequences (see above), the one identified as *L. nodulosus* was clearly assigned to *T. nodulosus* from the Raupach et al. [25] dataset. The two *T. arcuatus* sequences clearly differed from these and from each other. *Ligidium germanicum* was recorded throughout Germany and various Austrian states, whereas *L*. cf. *germanicum* was recorded only in Austria (Styria and Carinthia) and not in Germany.

For the two *L. germanicum* mOTUs, we compared the morphology of the endopod of pleopod II – a morphological character commonly used for the delimitation of *Ligidium* species – to preliminarily assess whether these may represent separate species.They showed minor but consistent differences in the shape of the tip of the pleopod II endopod, with *L.* cf. *germanicum* featuring an additional protrusion or hump in the center of the tip, while *L. germanicum* has a smoothly rounded central part (Fig 4).

The basal nodes were poorly resolved with very low bootstrap support (Fig. 3), which is expected for a COI-based phylogenetic analysis across such divergent taxa. It is noteworthy that the genera *Porcellio* and *Haplophthalmus* were non-monophyletic. For *Porcellio*, the species appeared at very different positions in the phylogenetic tree and for *Haplophthalmus Calconiscellus* was nested within *Haplophthalmus* and *H. danicus* clustered with *Tachysoniscus* (but with noo support).

As mentioned above, our study included historic specimens, which were quite different in terms of usability. From 80 historic specimens 19 COI sequences were obtained – of 12 specimens COI was obtained via Sanger sequencing of the mini-barcode and of 12 specimens via Illumina sequencing (Table 2). Of five of these specimens, Sanger as well as Illumina sequences were obtained. In these instances, Sanger-based and Illumina-based COI sequences were fully identical. Three specimens belonged to species for which no other COI data was available (S1 Fig), in the other cases, the historic sequences were highly similar or even identical to those of fresh specimens. Initial test runs had shown that full-length COI could not be PCR amplified in these specimens. A test run using COI mini-barcodes yielded PCR products for four of 21 historic DNA extracts, which increased to seven out of 21 (including the same four which had been positive before) when the DNA extract was treated with the DNA repair kit prior to PCR. Therefore, in the following, all further PCRs for Sanger sequencing and all library preparations for Illumina sequencing utilized the repaired DNA extracts. During Illumina library preparations, test runs indicated that G-tailing yielded slightly higher amounts of library in the desired fragment sizes (~200–650 bp), than libraries generated without G-tailing. Therefore, the majority of libraries were generated with the G-tailing. Of 33 Illumina sequenced libraries, 25 had been G-tailed of which 10 yielded COI (40%) and eight had not been G-tailed of which two yielded COI (25%). It should be noted that only libraries with sufficient concentration after PCR had been sequenced, four libraries were discarded before sequencing.

**Table 2:** Overview of molecular methods and sequencing success of historic specimens. For each specimen, the NHM-ZOO-CR- is provided. Methods which resulted in a COI barcode are marked by a “+”, methods that were employed for a specimen but did not result in a DNA barcode are marked by a “-”; empty cells indicate combinations of methods that had not been tested for the respective specimen. The kit that was used for the library preparation (following FFPE-repair and/or G-tailing) are indicated in the last column: ULTRA II = NEBNext® Ultra™ II DNA Library Prep Kit for Illumina; FS = NEBNext® Ultra™ II FS DNA Library Prep Kit for Illumina (this kit includes an additional fragmentation step).

ID	Sanger: FFPE-repair	Illumina: FFPE-repair/G-tailing	Illumina: no FFPE -repair/G-tailing	Illumina: no FFPE repair/no G-tailing	Illumina: FFPE-Repair/no G-tailing	Kit Illumina library prep.	number Illumina reads before quality trimming	number Illumina reads after quality trimming
12196	–	–				FS	510.738	20.510
12212	+	–			–	Ultra II	118	3
12234	–	–			–	Ultra II	2.817.539	6.077
12422	+	+				FS	1.601.729	1.383.154
12786	–	–				FS	1.723.599	226.717
13125	–				+	FS	13.717.706	12.911.144
13133	–			–	–	FS	41.047.648 (repaired)	39.626.244 (repaired)
13775	+							
13863	–	–				FS	976.230	460.222
13917	–	–				FS	675.265	41.533
13945	–	–				Ultra II	NA	NA
14102	+	–				Ultra II	9.699.872	7.438.541
14264	+							
14367	–	–	+			FS	3.082.405	93.003
14339	+							
14401	–		+			FS	1.299.852	508.650
14958	–	–				FS	882.828	16.984
15059	+	“- (FS)”			+ (Ultra II)/- (FS)	FS/Ultra II	47.339.380 (no G-tailing) 2.221.738 (G-tailing)	44.251.370 (no G-tailing) 1.153.277 (G-tailing)
15504	–	+				FS	2.221.738	1.153.277
15511	–	–				Ultra II	4.700.326	8.136
15811	+	–				Ultra II	17.111.788	4.625.694
15893	+							
15970	+							
16057	–	–			–	Ultra II	2.470.989	13.115
16299	–	+				FS	14.003.823	11.159.367
16383	–	+				FS	2.042.490	1.856.158
16964	–	–				FS	1.258.249	39.873
17346	+	+				FS	1.374.139	172.371
17604	+	+				FS	859.226	770.226
17628	–	–				FS	1.748.290	90.347
22134	–	–				FS	NA	NA
8841	–	–				FS	NA	NA
9611	–	–				FS	2.972.488	971.275
9682	–	+				FS	30.208.122	23.061.145
9711	–	+				FS	1.590.388	1.386.312

COI sequences derived from Illumina data were usually longer than the mini-barcodes (often spanning the complete studied COI fragment). Shorter Illumina-derived COI sequences, however, were not always overlapping with each other, being either from the 5-prime or 3-prime end. This greatly reduced their usability as they could not be fully compared to each other or to existing data (e.g., the sequence of *T. arcuatus*).

## Discussion

### Species diversity and DNA barcoding

Of the 64 nominal (sub)species known from Austria, we were able to provide DNA reference barcodes for 49 (plus one of an unidentified *Trichoniscus* species). This corresponds to 76% of the known Austrian Oniscidea fauna.

In his latest review of the Austrian Oniscidea, Strouhal [14] listed 64 species and subspecies. However, he omitted *Haplophthalmus montivagus* (see also [16,22] and included *T. illyricus* twice: on the one hand *T. illyricus* (see [12]) and on the other *T. illyricus carinthiacus* (see [14]). According to Schmölzer [16], the nominate form (which would be *T. illyricus illyricus*) does not occur in Austria. Comparison to Schmalfuss [9] and WoRMS (accessed 06/2025) showed that three of the previously listed species and subspecies are now considered junior synonyms of other species occurring in Austria: *Androniscus stygius tschameri* and *Androniscus stygius kesselyáki* (both now *A. stygius*), *Tracheoniscus pseudoratzeburgi apenninorum* (synonym of *T. arcuatus*), and *Protracheoniscus amoenus amoenus* (synonym of *P. politus*). In addition, some of the subspecies have now been raised to species level.

The following three nominal species have been newly reported for Austria since Strouhal [14] and Schmölzer [16]: *Armadillidium arcangelii* (see also [23]), and *Philoscia affinis affinis* and *P. muscorum muscorum* in this study. In total, the number of nominal Austrian Oniscidea species and subspecies remains unchanged at 64 (Table 1), although the species composition has changed slightly.

Of the newly reported species, *A. arcangelii* is not native to Austria and has been introduced by human activities; so far only a single localized population in Vienna is known [23]. The presence of the two *Philoscia* species is surprising as no *Philoscia* species had been included in any of the previous species lists for Austria [12,14,16]. *Philoscia muscorum muscorum* was found only in western and northwestern Vienna and all individuals exhibited the identical COI haplotype, which is also the most common haplotype in Germany (see S2 Fig; [25]). This may indicate that also *Philoscia muscorum muscorum* is an introduced species, though range expansion or simply being missed in previous studies is a possibility. Conversely, *P. affinis affinis* had a wider geographic distribution in Austria with records from several localities in Lower Austria and Styria and it includes a larger number of COI haplotypes. This rather suggests it is native in Austria. The historic collection of the Natural History Museum Vienna holds a very detailed and extensive Oniscidea collection from Austria. In total, seven lots of Austrian *Philoscia* specimens from Vienna, Carinthia, Tyrol and Lower Austria dating back to as early as 1890 are present. As all were females, an unambiguous identification was not possible, though some had been historically identified as *P. muscorum*. Although this underpins the long-term presence of *Philoscia* in Austria, the number of historic records is relatively small compared to many other species; whereas *Philoscia* was quite commonly encountered in our study. Maybe it has become more common in recent years. Why these historic records were not considered by previous authors (e.g., [12,14,16]) and why these species were never encountered by them remains an open question.

Some of the 49 (sub)species, for which DNA barcodes were obtained, are very rare and difficult to collect, including *Mesoniscus alpicola*, which is found exclusively in caves, and *Oroniscus mandlii*, which is known from a single mountain and of which only two specimens had been previously collected [13]. These newly generated DNA barcodes are an important addition to the Austrian Barcode of Life (ABOL) initiative (www.abol.ac.at; [8]). Until now, however, there had been little data on isopods. The remaining ~ ¼ of species, for which we were not able to produce DNA barcodes, belong mainly to the small-bodied Trichonsicidae including several in-soil or subterranean species. Also, species and populations from western and/or northern Austria were underrepresented, as our study was biased towards eastern and southern Austria (especially Vienna, Lower Austria, Styria and Carinthia).

The presence of multiple, genetically very clearly differentiated mOTUs in several nominal species suggests the presence of additional species. Reported intraspecific COI distances for Oniscidea are usually below 4% (e.g., [56,57]) or below 6.5% [58] with interspecific distances usually exceeding 9% [56–60]. However, minimum interspecific distances may be underestimated, as several of these studies did not necessarily focus on putative sister species. Data from marine isopods suggests intraspecific distances of <6% and interspecific distances exceeding 10% [61–65].

With the data available, it is not possible to unambiguously decide which of the mOTUs represent actual species and which are rather genetically divergent intraspecific lineages. The comparison to the previous studies and the divergence patterns in the studied species, suggests that our most conservative estimate (ASAP9.8) probably underestimates actual species numbers. However, even under this conservative approach several species are split into multiple mOTUs, which most likely represent cryptic species. This includes the ant-associated species *Platyarthrus hoffmannseggii,* the cave-living *M. alpicola* and three *Trichoniscus* species (*T. noricus*, *T. pusillus* and *T. steinboecki*) as well as *L. germanicum.* For the latter, our preliminary morphological data on the male pleopod II endopod further supports the distinction between the two *L. germanicum* mOTUS (here termed *L. germanicum* and *L.* cf. *germanicum*). The respective morphological differences may be minor, but consistent with differences observed among other *Ligidium* species [24]. Together with the 18.5–20.0% *p*-distance in COI, this strongly suggests that these represent reproductively isolated species. The mOTU identified as *L. germanicum* probably represents the “true” *L. germanicum* as its distribution spans southern Germany, where the species was first described from. We hesitate to formally describe *Ligidium* cf. *germanicum* as a formal species herein as this would require a larger and more comprehensive sample of the species and a more in-depth morphological characterization.

Conversely, the two subspecies *P. fiumanum fiumanum* and *P. fiumanum salisburgense* were lumped together at a 9.8% threshold as were *A. opacum* and *A. carniolense*, but they were separated in the partitions with thresholds of 4.8% and 6.2%. Together with their morphological differences, this suggests that these (sub)species are indeed separate species. It further strengthens the assumption that several of the other mOTUs separated by lower threshold values represent distinct species as well. Our least conservative estimates (ASAP4.8 and even more BINs) probably overestimate species diversity and we assume that the actual species diversity is closer to ASAP6.2. Of course, no universal barcoding threshold exists and each species (or mOTU) pair needs to be assessed independently in an integrative framework including morphological and nuclear data. This requires extensive revisions including material from other countries as some of these mOTUs may correspond to previously synonymized species or subspecies. For this reason, we do not propose any taxonomic changes herein. The example of *P. collicola* highlights that for some presumably well-known species the extent of morphological variability is not fully understood, which in turn may lead to erroneous species identifications. Integrative approaches that document the morphological variability across the species’ geographic range are needed. This may be particularly important for the small-bodied Trichoniscidae where we document several examples of putative cryptic species, showing the need for a more thorough revision. Similarly, the non-monophyly of the genera *Porcellio* and *Haplophthalmus* may indicate the need for further systematic revisions. However, our phylogenetic analysis was based on a single, fast evolving gene and more detailed analyses are required before any systematic and taxonomic changes are proposed. In summary, our results indicate the presence of multiple hitherto undescribed species, suggesting that the diversity of terrestrial isopods in Austria may be even higher than the currently accepted 64 species.

### Museomics

Including historic museum specimens in DNA barcoding studies has many advantages. They can fill gaps in DNA barcoding reference databases – which is particularly important for rare, difficult to access and collect, threatened, protected or even extinct species – and allow the integration of expert knowledge of previous experts, by integrating specimens identified by the respective historic experts. The gold standard would, of course, be the integration of historic type specimens. Due to their high scientific value, the latter can be difficult for many small-bodied taxa, as DNA extraction is often at least partly destructive (see also [45]). For this reason we did not attempt sequencing historic types herein. Our results underline the overall value of such museomic (*sensu* [44]) approaches. DNA barcodes of three species were only obtained from historic museum specimens and these species would not have been represented otherwise.

A variety of different museomic approaches exist. We tested and compared Sanger-based as well as Illumina-based approaches. For Sanger-based approaches, targeting mini-barcodes (instead of longer fragments) and the treatment of the DNA with a DNA repair kit prior to PCR greatly increased PCR amplification and sequencing success. This is probably the most effective approach in terms of costs and lab work to generate DNA barcodes of historic specimens and could probably also be integrated in large-scale sequencing approaches [66,67]. Higher success rates of mini-barcodes compared to full-length barcodes had been demonstrated before, sometimes with success rates close to 100% (e.g., [68–70]). The higher overall success rates in those studies compared to ours, may be due to differences in fixation, preservation and storage of the specimens. DNA quality appears to degrade faster in ethanol-fixed specimens than in dried specimens (e.g., [42]) and some of the specimens studied by us were probably fixed in formalin. Unfortunately, this was not well documented in the past. Of course, mini-barcodes contain less information compared to full length-barcodes due to their shortness, but their overall effectiveness in species delimitation has been demonstrated [69,71] and we would argue that a short barcode is preferable to no barcode, though we would always try to obtain full barcodes if possible. We do not know which of the repair steps included in the DNA repair kit is responsible for the increased PCR amplification success. Probably it is either the repair of the deaminated bases (which would have prematurely terminated the PCR amplification) or the repair of single-stranded nicks, as these nicks would result in much shorter fragments when the DNA is denatured during PCR.

Illumina-based target capture was also successful in generating high quality COI sequences, with the advantage that these COI sequences were usually (but not always) longer than the mini-barcodes and, of course, also other genetic markers may have been retrieved if wanted. However, the overall efforts in costs and lab work are higher compared to Sanger-based mini-barcodes, especially when G-tailing or commercial kits are included in the library preparation. We observed only minor differences in success rates between Illumina libraries with G-tailing compared to those without. Maybe other methods developed for converting ssDNA into dsDNA are more effective (e.g., [72–74]. Despite the higher effort compared to Sanger-based approaches, it is worthwhile to employ Illumina-based approaches for scientifically valuable specimens for which Sanger-based approaches failed or if genetic data beyond DNA barcodes is desired.

In summary, our study provides important reference COI barcodes for the Austrian terrestrial isopod fauna. These will be relevant for future metabarcoding-based biodiversity and monitoring studies of soil organisms. Especially, for many of the small-bodied taxa such reference data has been missing. Our results also highlight the potential of cryptic diversity in this group and the utility of museomic approaches in DNA barcoding approaches.

## Supporting information

S1 Fig Uncollapsed maximum likelihood phylogenetic tree of Austrian Oniscidea based on COI. For each specimen, the respective collection number and the Austrian state where it was collected are provided. Bold font indicates sequences shorter than 350 bp, red font indicates historic specimens (here, the historic and often outdated species names are provided as well). Only bootstrap support values >50% are shown, support values for minor intraspecific nodes are usually not shown. Vertical bars show mOTUs suggested by the three ASAP partitions as well as BINs assigned by BOLD. Each bar represents one mOTU, bars were alternately colored black and grey for better visualization. It should be noted that for some specimens with too short sequences, no BIN was assigned, no bar is shown in these instances.
(EPS)

S2 Fig Maximum likelihood phylogenetic tree including Austrian Oniscidea and the data from Raupach et al. [25] based on COI. For each specimen, the respective collection or GenBank number as well as country and state where the specimen was collected are provided. Bold font indicates sequences shorter than 350 bp, red font indicates historic specimens (here the historic and often outdated species names are provided as well). Only bootstrap support values >50% are shown, support values for minor intraspecific nodes are usually not shown.
(EPS)

S1 Table Specimen details. For each specimen details are provided such as locality information, collector, collection date, inventory number and BOLD numbers. The dataset is also available from BOLD: dx.doi.org/10.5883/DS-ISOBA.
(XLS)
